# High Individuality of Respiratory Bacterial Communities in a Large Cohort of Adult Cystic Fibrosis Patients under Continuous Antibiotic Treatment

**DOI:** 10.1371/journal.pone.0117436

**Published:** 2015-02-11

**Authors:** Rolf Kramer, Annette Sauer-Heilborn, Tobias Welte, Ruy Jauregui, Ingrid Brettar, Carlos A. Guzman, Manfred G. Höfle

**Affiliations:** 1 Department of Vaccinology and Applied Microbiology, Helmholtz Centre for Infection Research, Braunschweig, Germany; 2 Department of Pneumology, Hannover Medical School, Hannover, Germany; 3 Department of Medical Microbiology, Helmholtz Centre for Infection Research, Braunschweig, Germany; 4 German Centre for Infection Research and German Centre for Lung Research, Hannover, Germany; University of Western Australia, AUSTRALIA

## Abstract

**Background:**

Routine clinical diagnostics of CF patients focus only on a restricted set of well-known pathogenic species. Recent molecular studies suggest that infections could be polymicrobial with many bacteria not detected by culture-based diagnostics.

**Methodology and Principal Findings:**

A large cohort of 56 adults with continuous antibiotic treatment was studied and different microbial diagnostic methods were compared, including culture-independent and culture-based bacterial diagnostics. A total of 72 sputum samples including longitudinal observations was analysed by 16S rRNA gene sequence comparison. Prevalence of known pathogens was highly similar among all methods but the vast spectrum of bacteria associated with CF was only revealed by culture-independent techniques. The sequence comparison enabled confident determination of the bacterial community composition and revealed a high diversity and individuality in the communities across the cohort. Results of microbiological analyses were further compared with individual host factors, such as age, lung function and CFTR genotype. No statistical relationship between these factors and the diversity of the entire community or single bacterial species could be identified. However, patients with non-ΔF508 mutations in the CFTR gene often had low abundances of *Pseudomonas aeruginosa*. Persistence of specific bacteria in some communities was demonstrated by longitudinal analyses of 13 patients indicating a potential clinical relevance of anaerobic bacteria, such as *Fusobacterium nucleatum* and *Streptococcus millerii*.

**Conclusions:**

The high individuality in community composition and the lack of correlation to clinical host factors might be due to the continuous treatment with antibiotics. Since this is current practice for adult CF patients, the life-long history of the patient and the varying selection pressure on the related microbial communities should be a focus of future studies and its relation to disease progression. These studies should be substantially larger, providing more molecular information on the microbial communities complemented by detailed genetic assessment of the host.

## Introduction

Cystic fibrosis (CF) is regarded as the most common genetic disorder in the Caucasian population and respiratory infections of CF patients are the leading cause of death [1]. CF affects the ion and water transport of cells, leading to viscous mucus and disturbed body functions, such as the clearance mechanism of the lung [2, 3]. As a consequence, particular bacteria cause airway infections in CF patients with recurrent exacerbations and entail immune responses which in turn are considered to be responsible for the majority of irreversible lung damages [4]. Since improved diagnostics and health management led to an increasing life expectancy of CF patients, it is becoming apparent that disease manifestations and infections are changing with age resulting in new challenges for the treatment of adult CF patients [1, 5].

Conventional clinical microbiology is based on cultivation focussing on a restricted set of key pathogens but culture-independent methods, mostly based on 16S rRNA gene analysis, revealed a broad spectrum of bacterial species associated with CF which were previously under-diagnosed or not detected at all [6, 7]. Hereby, compositional analyses of microbial communities are of growing interest [8] and in particular, next generation sequencing (NGS) technologies offer great potential to explore the microbiome of the respiratory tracts. Currently, a complex picture of bacterial communities in the respiratory tract of CF patients is developing with emerging pathogens, anaerobic species and polymicrobial interactions [4, 9, 10, 11].

In this context two classical dictums about microbial distribution and pathogenicity have been critically reconsidered in recent years. On the one hand, Lourens Baas Becking’s “everything is everywhere but the environment selects” [12] is apparently transferable on the microbial communities in the CF airways in which certain ecological conditions promote the growth of different microorganisms. On the other hand, Robert Koch’s dictum assuming that one microorganism causes one disease is currently under debate and could be replaced by a more comprehensive view of the whole bacterial community involving inter-species interactions resulting in different pathogenic potential of certain microbial assemblages [13, 14]. In this context, recent studies further indicate that our understanding of the underlying mechanisms and the CF airways is fragmentary whereas the key pathogens, like *Pseudomonas aeruginosa* and *Staphylococcus aureus* are well identified [5], the presence and conceivable interactions with other bacteria in the respiratory tract are still unclear [15]. To this end, the study of the bacterial community composition of individual CF patients is needed and its relation to host factors, like lung function and genotype of the CFTR gene, should be explored.

The aim of this study was to determine the individual bacterial communities of airways from CF patients, to assess their relation to host factors in a cohort of adult CF patients, and determine their dynamics in individual patients. Therefore, bacteria in sputum samples of a large cohort of adult CF patients were identified using three independent microbiological methods, i.e. classical cultivation, molecular fingerprinting and deep sequencing (NGS). Hereby, ‘relative abundances’ are used to illustrate the proportion of specific bacteria in the individual community of each sputum sample and ‘prevalence’ is used to illustrate the occurrences of these bacteria in the entire cohort. Overall, high individuality of bacterial communities was observed for adult CF patients with no obvious correlation to the actual lung function or the genotype of the CFTR gene of the host. This observation might be due to the continuous treatment with antibiotics of the adult CF patients. Longitudinal analyses revealed that anaerobic and infrequent bacterial species are not only transient but stable elements of individual bacterial communities. Our results emphasise the relevance of polybacterial assemblages, including anaerobic bacterial species with moderate prevalence and high relative abundance in the studied CF cohort of adult patients under continuous antibiotic treatment.

## Results

### Microbial community profiling of individual samples

Microbial profiling of 72 sputum samples was performed to gain insights in the role of bacterial species for CF airway communities. Microbial community profiling consists of two elements: i) identification of the bacteria present and ii) quantification of the abundances of the single species Operational Taxonomic Units (OTUs). Both of the culture-independent approaches used, SSCP fingerprints and deep sequencing, allow a straight forward determination of relative abundances of bacterial species as detailed in the materials and methods. In order to assess abundances of bacterial species in the cohort during the sampling period of 2 years, deep sequencing was compared with molecular fingerprinting and culture-based diagnostics routinely performed in clinical microbiology, respectively. Using bar-coded Illumina deep sequencing and conventional molecular fingerprinting by SSCP electrophoresis, culture-independent diversity analysis of bacteria in the CF cohort was performed based on the definition of OTUs. A high degree of agreement is demonstrated for the prevalence of bacteria on the genus level assessed by both methods; hereby a minimum relative abundance of ≥ 5% in individual samples was used to compare the results of deep sequencing and SSCP fingerprinting that both have a lower but different limit of detection (Fig. A in S1 File). Some discrepancies in taxonomic resolution were particularly observed for the genus *Streptococcus*; however, in both approaches alpha-hemolytic streptococci and the anaerobic *Streptococcus millerii* group could be confidently distinguished (Fig. B in S1 File). The relative abundances of the most dominant bacteria in each individual sample assessed by both approaches were highly similar irrespective to taxonomic affiliation of the species (Fig. C in S1 File).

The reproducibility of our molecular approach was further analyzed by a statistical assay enabling a direct comparison of the community profiles from the entire cohort and a high degree of agreement for the communities assessed by both approaches was revealed. The strong correlation is indicated by a rank correlation value (Spearman’s rho) of 0.816 (P-value = 0.01). This statistical method calculated a correlation coefficient by testing similarity of compositional data sets for all 72 sputum samples assessed by both molecular approaches (SSCP and NGS) (Table A in S1 File). The high correlation value obtained for both approaches indicates, despite different taxonomic resolution, the reliability and robustness of NGS and SSCP in microbial community profiling. The community profiles assessed by deep sequencing (NGS) were chosen to be applied to further analysis because of the better quantitative resolution.

Microbial profiling revealed that community composition in the sputum samples was ranging from highly diverse communities to almost a monoculture of an individual bacterial species. To assess alpha diversity of individual communities we calculated the richness and the Shannon diversity index based on the NGS data using OTUs with a relative abundance of larger than 0.5% (Table B in S1 File). Richness ranged from 1–22 OTUs per sample with an average of 8.6. The Shannon diversity index ranged from 0.07 to 2.03 averaging at 1.13. Comparison of richness and the Shannon diversity index resulted in a good logarithmic correlation with a regression coefficient of r^2^ 0.834 as expected (Fig. D in S1 File). Furthermore, the Shannon index, used as a measure of alpha diversity did not show an obvious correlation with age or lung function, i. e. FEV_1_ value, of the patients (Fig. E and F in S1 File).

**Fig 1:**
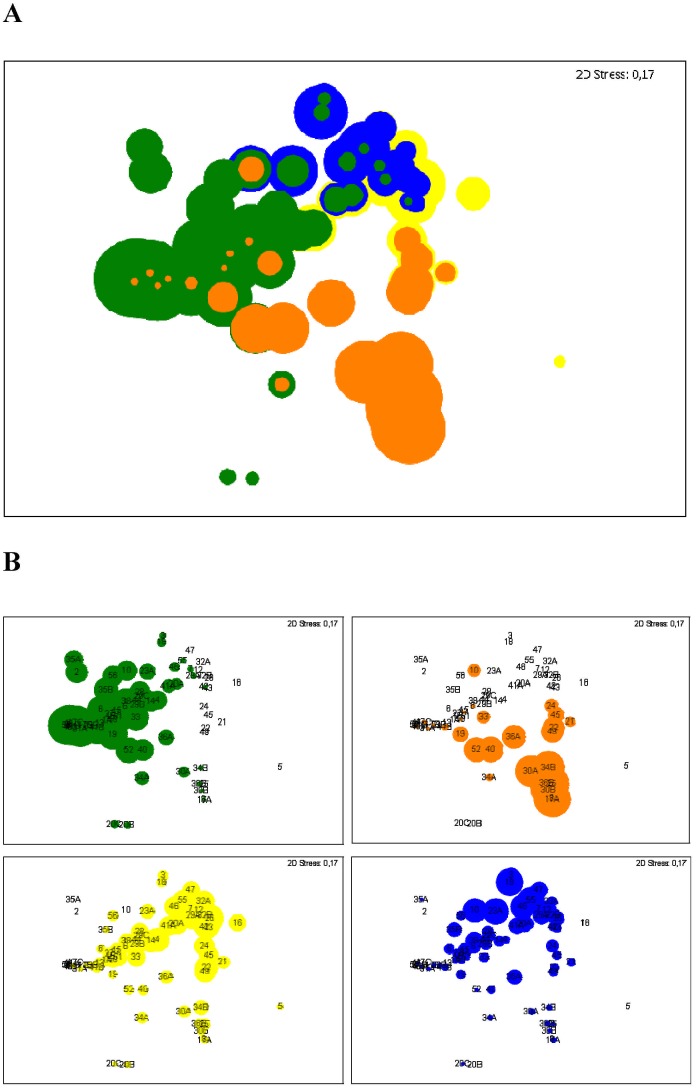
Multidimensional Scaling (MDS) plots from bacterial community composition observed in 72 sputum samples. Bubbles indicate relative abundances of the 4 most prevalent OTUs in the cohort: *P*. *aeruginosa* in green, *S*. *aureus* in orange, alpha-hemolytic *Streptococcus* (*Streptococcus* -1) in yellow, *R*. *mucilaginosa* in blue. 2D Stress values are given in each plot and reveal moderate stress. **(A)** Merged bubble plots from all four dominant OTUs, coloured bubbles indicating the individual abundances. *P*. *aeruginosa* as well as *S*. *aureus* have priority, whereas the abundances for *R*. *mucilaginosa* and alpha-hemolytic *Streptococcus* are in the background. (**B)** Individual MDS plots for each dominant OTU. Numbers indicate individual patients. Letters are according the order of sputum collection.

Multidimensional Scaling (MDS) plots were used to compare individual communities and their most abundant bacterial species. In these MDS plots beta diversity is assessed by comparing the samples according similarity values that result from the pairwise comparison of each sample in similarity matrices. The more similar the community compositions of each sample, the more closely they are together in a MDS plot. The ordination of the samples (Fig. 1A) indicates the high diversity of the community composition in the samples with a high individuality by the distribution of the individual communities across the entire plot. Nevertheless, all samples exhibited at least one of the previously identified most important OTUs, such as *P*. *aeruginosa*, *S*. *aureus*, *Rothia mucilaginosa* and alpha-hemolytic streptococci as illustrated in Fig. 1B.

**Fig 2:**
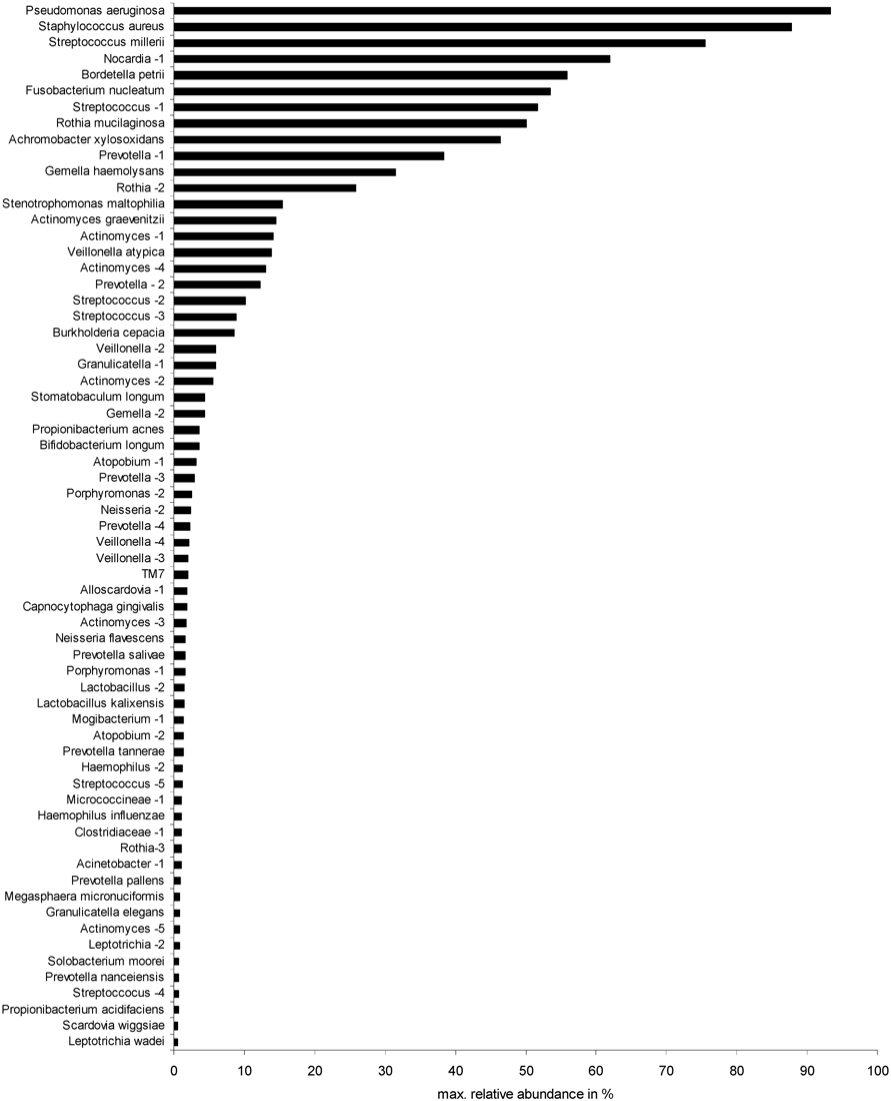
Maximum relative abundances of the bacterial OTUs determined in individual sputum samples. Individual relative abundances were determined by deep sequencing. OTU identification was achieved by comparison with SSCP sequences.

A broad spectrum of other bacterial species was observed in the sputum samples (Table C in S1 File). Maximum relative abundances of the bacteria in individual sputum samples observed by deep sequencing are given in Fig. 2 and show a few dominant species together with a long tail of bacteria detected in low relative abundances. Besides key pathogens, such as *P*. *aeruginosa* or *S*. *aureus*, also infrequently detected bacteria like *Nocardia* sp. or *Bordetella petrii* and anaerobes like *F*. *nucleatum* and the *S*. *millerii* group were highly dominant, i. e. relative abundances above 50%, in individual communities, indicating a high colonization ability of these species. Relative abundances above this threshold were observed for *R*. *mucilaginosa* and *A*. *xylosoxidans*.

### Comparison of the bacterial community composition and host factors

The relationships between bacterial communities, host factors and clinical aspects were analyzed. In this respect we used four relevant clinical features: i) the age of the CF patients, ii) the type of antibiotic treatment, iii) the lung function represented by the FEV_1_ values, and iv) the genotype of the CFTR gene of the CF patients.

**Fig 3:**
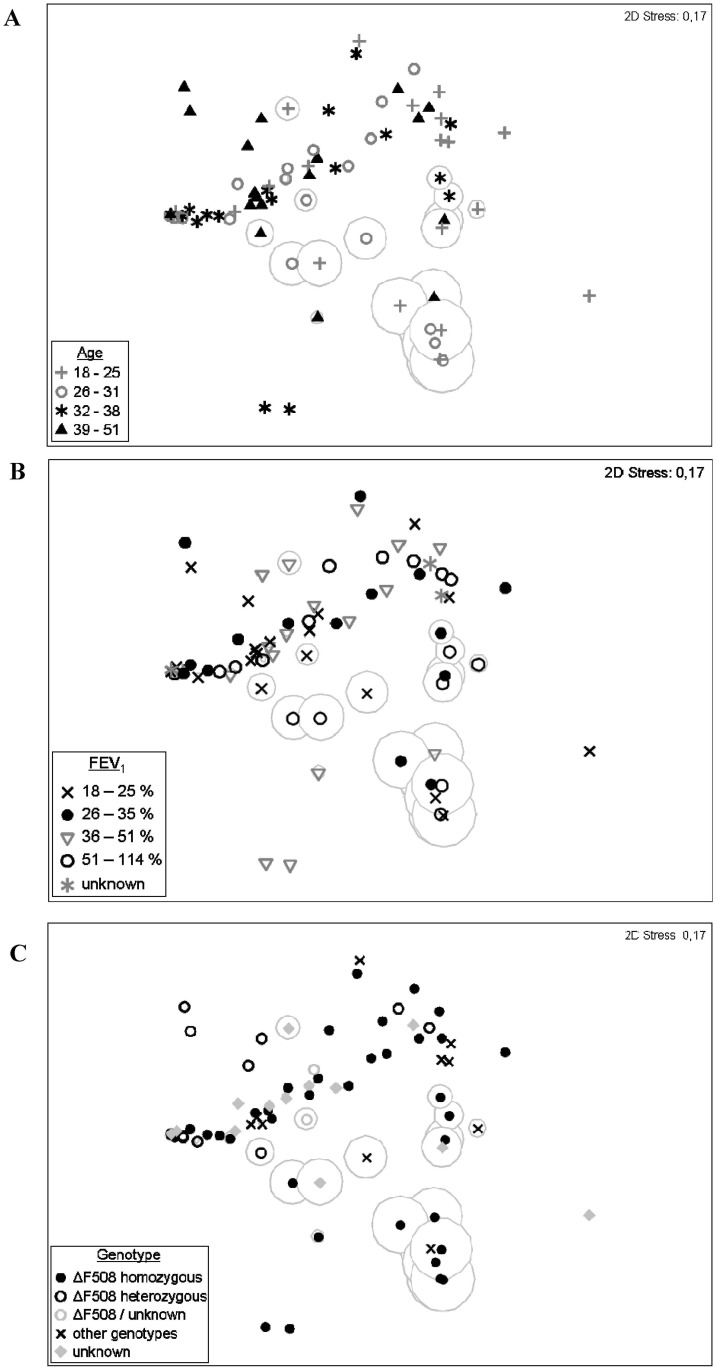
Multidimensional Scaling (MDS) plots from bacterial community composition observed in 72 sputum samples with superimposed host factors. For a better orientation in the plot, relative abundances of *S*. *aureus* are indicated as in Fig. 1B (orange circles). **(A)** Age of patients superimposed on the previous MDS plot calculated for the community compositions of 72 sputum samples. Four age classes were defined for the cohort according to the statistical quartils. Symbols indicate the age of the patient at the time point of sputum collection. **(B)** Lung function of patients superimposed on the previous MDS plot defined for the community compositions of 72 sputum samples. Four classes of lung functions were calculated according to the statistical quartils of FEV_1_ values measured for the patients on the day of sputum collection. Symbols indicate the four classes and samples without associated FEV_1_ values. **(C)** CFTR mutations of patients superimposed on the previous MDS plot calculated for the community compositions of 72 sputum samples. Three classes of identified genotypes were determined for the cohort. Partly or completely unknown genotypes are accordingly labelled representing a fourth class.

In order to examine any correlation between the overall community composition and the patient’s age, the cohort was divided into four age classes according to the corresponding statistical quartils. Symbols for each age class were superimposed in the previous MDS plot calculated for the community composition of all 72 sputum samples (Fig. 3A). No correlation was observed between age of the patient and the four previously defined categories of bacterial communities. Patients with a community composition dominated by the major CF pathogens *P*. *aeruginosa* and *S*. *aureus* were distributed equally in all age classes as well as patients with high relative abundances of *R*. *mucilaginosa* and *Streptococcus*-1. In more detail, the relationship of *P*. *aeruginosa* and age is given (Fig. G in S1 File). Samples were ordered by the age of patients at the time point of sputum collection and compared with the individual relative abundances of *P*. *aeruginosa*. Again, no correlation was observed. Although most samples without presence or with low relative abundances (≤ 5%) of *P*. *aeruginosa* were observed for patients with ≤ 25 years, similarly low abundances were also detected in several samples of other age classes. Likewise samples from patients of all ages exhibit communities dominated by *P*. *aeruginosa*. In summary, a tendency for young adult CF patients to exhibit less frequently communities with highly abundant *P*. *aeruginosa* could be shown but exceptions in individual cases were observed.

The antibiotic treatment, as detailed in Materials and Methods, was compared with the bacterial community structure of individual patients using MDS plots. For these statistical analyses the antibiotic treatment was categorized in three types of application: i) inhalation, ii) oral, and iii) intravenous application within the past 14 days before the sputum sample was obtained. The MDS plots did not show a significant correlation between bacterial community structure and type of antibiotic treatment (Fig. H in S1 File). We also tried to find correlations to the specific antibiotic used for the treatment with the same negative result. Therefore, we conclude that there was no obvious relationship of the bacterial community structure with this host factor.

For the study of correlations between the overall community composition and the lung function of the patients, the cohort was divided into four classes according to the statistical quartils of the FEV_1_ values of all patients. Symbols for each class were superimposed in the previous MDS plot calculated for the community composition of all 72 sputum samples (Fig. 3B). A median predicted FEV_1_ of 35% was calculated for the cohort and patients with strongly reduced lung functions (≤ 25%) were observed in all four previously defined categories of bacterial communities. Likewise, relatively high FEV_1_ values of ≥ 51% were observed for patients with communities dominated by the major CF pathogens *P*. *aeruginosa* and *S*. *aureus*. Further demonstrated were the relationship between *P*. *aeruginosa* and the lung function: Samples were ordered by relative abundances of *P*. *aeruginosa* and compared with the predicted FEV_1_ values (Fig. I in S1 File). No clear correlation was observed between the lung function and the relative abundance of *P*. *aeruginosa* and other key taxa.

Finally, correlation analyses were further performed between the genotype of the CFTR gene of each patient and the corresponding community composition in the sputum samples (Fig. 3C). Patients were classified according to the genotypes: 41% were homozygous-ΔF508, 16% heterozygous-ΔF508, 16% had a non-ΔF508 mutation and for 27% of the patients the precise genotype was not or only partly determined. Neither of these genotype-classes correlated significantly with communities dominated by *P*. *aeruginosa*, *S*. *aureus*, *R*. *mucilaginosa* or *Streptoccocus*-1. However, the class of patients with non-ΔF508 mutations was composed of diverse genotypes. In only three samples from patients out of this class, the community was dominated by *P*. *aerugionsa* with relative abundances ≥ 50% and in seven samples the communities exhibited only low abundance of *P*. *aeruginosa* (≤ 5%). Overall, the high relative abundances of *P*. *aeruginosa*, *S*. *aureus*, *R*. *mucilaginosa* and *Streptoccocus*-1 in sputum samples could not be clearly correlated with any clinical parameter or host factor of the CF patients.

### Community dynamics of detected bacteria

**Fig 4:**
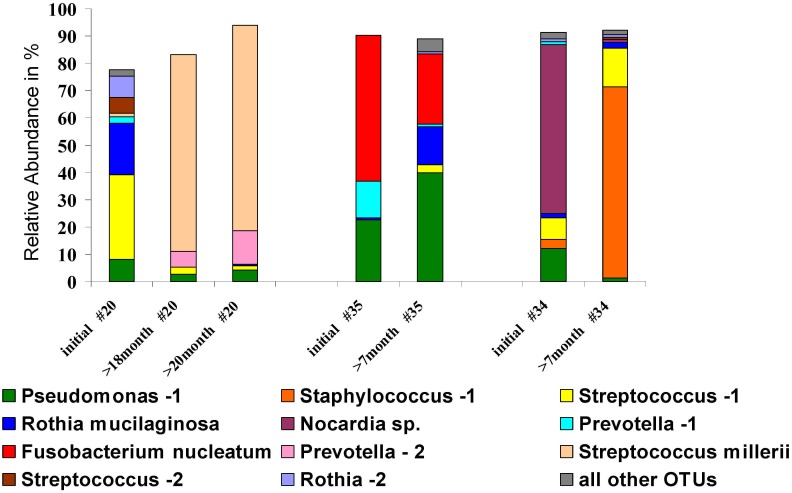
Dynamics of community composition in three representative individuals (patient #20, #35 and #34) from the sub-cohort of patients that provided samples twice or three times. Each OTU is indicated by a specific colour and further identified in the figure legend. CF patients are identified by numbers and sputum sample are shown in chronological order, hereby, time intervals to the initial sample are indicated in the plot. All other OTUs summarized in grey represent low abundant species not represented by the taxa indicate in the figure.

Presence of bacterial species in sputum samples collected over time can give insights into their clinical relevance. Therefore, dynamics of the community composition were studied in a sub-cohort of 13 CF patients. From each patient, sputum samples were collected within different time intervals to the initial sample. Considering the CF airways to be a challenging environment with permanent antibiotic application and immune responses from the host, dynamic changes in the microbial community compositions can be expected. However, variation of communities over time differed between the patients but a remarkable stability was observed for some communities (Fig. J in S1 File). Stability was considered if the relative abundance of single bacterial species is not changing substantially in two subsequent samples. Particularly, communities with high relative abundances of *P*. *aeruginosa* exhibited high stabilities over time, for the pathogen itself and similarly for the entire structure of the individual communities (see patients #29, 35, 6, 1, 31 in Fig. J in S1 File). Communities dominated by *S*. *aureus* were observed to be stable in some cases whereas complete changes in the composition were observed for others (see patients #36, 34, 30, 17 in Fig. J in S1 File). A specific focus was put on communities with high relative abundances of bacteria with unknown pathogenic potential, depicted in Fig. 4. The anaerobic species *F*. *nucleatum* and the *S*. *millerii* group dominated each of the communities in sputum samples of one patient collected at different time points (patient #35 and #20, respectively). For both bacteria a high stability with high relative abundances were observed over several months, indicating high colonization abilities in the CF airways. Before *S*. *millerii* was dominating the communities in the two samples taken after 18 and 20 months, a rather different community was observed in the initial sputum sample of patient #20. This may indicate a complete change in the associated environmental conditions in the respiratory tract of that patient which finally promote the growth of members of the *S*. *millerii* group. Species from the genus *Nocardia* were only detected in 4.2% of all sputum samples from the cohort. However, *Nocardia* sp. was observed in two samples collected from patient #34 at different time points. Whereas high relative abundance of 62% was observed in the initial sample, *Nocardia* sp. was still present in the sputum 7 months later, but only in low relative abundance (1.4%). Overall, we think that community dynamics of several months showed that there is certain stability in the community composition that is unlikely to be random.

### Prevalences of bacteria in the CF cohort

High abundances had been observed for the most prevalent genera *Pseudomonas* (NGS: 62.5%; SSCP: 68.1%), *Staphylococcus* (NGS: 25.0%; SSCP: 23.6%), *Streptococcus* (NGS: 66.7%; SSCP: 72.2%), *Rothia* (NGS: 50.0%; SSCP: 40.3%) and *Prevotella* (NGS: 18.1%; SSCP: 18.1%) with both molecular methods (Fig. A in S1 File). These genera reflect key pathogenic species, *P*. *aeruginosa* and *S*. *aureus*, as well as species considered part of the endogenous oral microbiome, like *R*. *mucilaginosa*, *Prevotella spp*., *Streptococcus parasanguinis* and *Streptococcus salivarius*. More *Proteobacteria* with pathogenic potential, such as *Achromobacter xylosoxidans*, *Stenotrophomonas maltophilia*, *Bordetella* sp. and *Burkholderia* sp. were detected in less than 10% of the samples.

**Fig 5:**
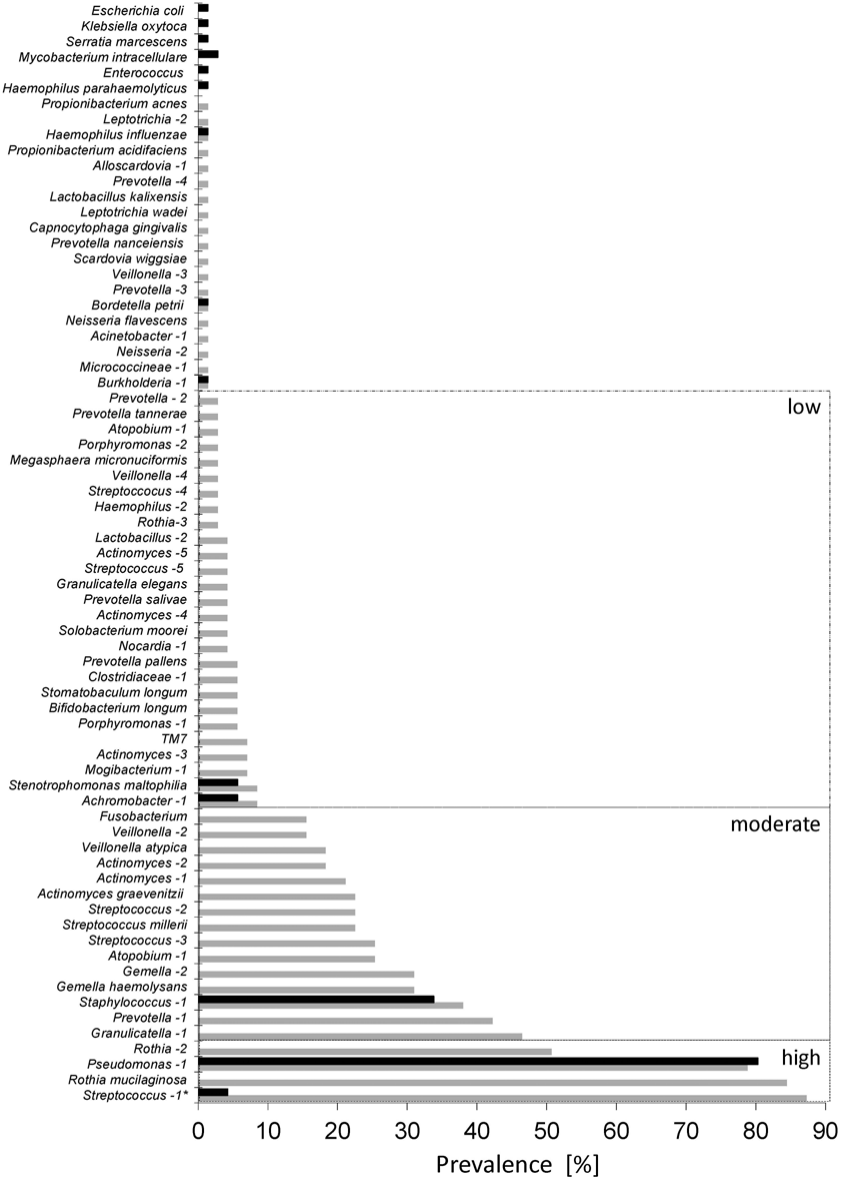
Comparison of prevalence of bacteria in the cohort identified by deep sequencing (NGS, grey bars) and cultivation for classical diagnostics (black bars). OTUs are listed on the left according their occurrence observed with deep sequencing. Bacteria present in >50% of all samples were categorized as highly prevalent, bacteria detectable in more than 10% of the cases were considered moderately prevalent and those observed in less than 10% of the cases were categorized as bacteria with low prevalence. Categorization follows detection rates by deep sequencing. For precise identification of bacteria see Table C in S1 File. Only 71 sputum samples were taken into account (no data from cultivation for sample #20B). * Streptococcus-1 was compared with alpha-hemolytic streptococci.

We assessed the prevalence of bacterial species in the entire cohort during the sampling period of 2 years using deep sequencing data. The prevalence of key pathogens assessed by deep sequencing was further compared with the reported results from the routinely performed culture-based diagnostics in clinical microbiology using standardized protocols. These standard cultivation procedures focus on the detection of *P*. *aeruginosa*, *S*. *aureus* or *B*. *cepacia*. Individual occurrence rates in clinical microbiology were compared with deep sequencing and revealed close correlations for all key pathogens indicating the reliability and importance of culture-independent methods. Results are summarized in Fig. 5. A high degree of congruence was observed for all pathogens, i.e. *P*. *aeruginosa* (NGS: 78.9%; cultivation: 80.3%), *S*. *aureus* (NGS: 38.0%; cultivation: 33.8%), *B*. *cepacia* (NGS: 1.4%; cultivation: 1.4%), *A*. *xylosoxidans* (NGS: 8.5%; cultivation: 5.6%), *S*. *maltophilia* (NGS: 8.5%; cultivation: 5.6%). However, the vast spectrum of bacteria associated with CF respiratory tracts was only revealed by deep sequencing (NGS). The total number of species reported by deep sequencing was five times higher than the one reported by cultivation. Besides those genera mentioned previously, a large number of bacteria with moderate or low occurrence were observed with deep sequencing due to the high quantitative resolution with a detection limit of at least 0.5% relative abundance. Bacteria detected only once and only by NGS were detected in low relative abundances close to the detection limit. A small number of species, such as *Klebsiella oxytoca*, *Escherichia coli* and *Serratia marcescens*, were only reported once by cultivation and might be considered as contaminants during cultivation. Interestingly, a substantial number of anaerobic bacteria, like the *S*. *millerii* group or *F*. *nucleatum*, were repeatedly detected with moderate occurrence rates but only by deep sequencing.

## Discussion

### Comparison of different diagnostic methodologies and abundances of aerobic species

Previously considered to be sterile, the lungs and their specific microbiome became a growing area of research interest due to classical and emerging new molecular techniques [16]. For children with CF, investigation of bronchoalveolar lavage fluid (BALF) by rRNA sequence analysis revealed a large number of bacteria not detected by cultivation but with potential clinical relevance [17]. In the present study, two molecular methods were applied to analyse microbial diversity and community composition in sputum samples. Assessment of microbial community profiles and bacterial prevalence in the CF cohort showed highly similar results by both molecular approaches despite differences in detection limit and taxonomic resolution (Fig. A in S1 File). Classical molecular fingerprinting methods, such as SSCP electrophoresis, allow confident elucidation of abundant bacteria, whereas the detection limit of NGS analyses includes also rare species but might have less taxonomic resolution depending on the read length of the technique [18]. Due to the high degree of agreement, the advantages of both methods were combined to elucidate the microbial communities in more detail. The key pathogens *P*. *aeruginosa* and *S*. *aureus* were, as expected, among the most prevalent bacteria in adult CF patients. The high prevalence of *Rothia* species and alpha-hemolytic streptococci was unexpected considering that only the species with a considerable relative abundance of 5% were taken into account. Both taxa are known to be part of the oral microbiome [19]. However, it has been shown previously that sputum samples are not extensively contaminated by species from the oral cavities [20]. Since these bacteria are known to be involved in oral biofilms, and the oral cavity is supposed to act as a ‘stepping stone’ for bacteria to colonize the airways in CF [32], they are likely to be also present in the lower respiratory tracts. The prevalence of other potential pathogens, like *S*. *maltophilia* and *A*. *xylosoxidans*, was similar to reported data from the literature [21, 22]. The reported data for the bacteria observed in CF patients is based on clinical diagnostics focusing on key pathogens of the phylum *Proteobacteria* and *S*. *aureus*. The high degree of agreement for the prevalence of these bacteria observed by comparison of molecular analysis and cultivation indicates the relevance of our findings and confirms the validity of culture based-diagnostics of known pathogenic bacteria.

### Abundances of anaerobic members of the bacterial community and their clinical relevance

The rather high prevalence and abundance of anaerobic bacteria, such as *F*. *nucleatum* and representatives of the *S*. *millerii* group, were unexpected. Recent publications revealed a broad spectrum of species associated with CF respiratory tracts [7, 9]. Accordingly, a high diversity of bacteria was detected by NGS with a detection limit of 0.5% relative abundance (Fig. 5). Anaerobic bacteria are of increasing interest in clinical research [10, 23] and moderate occurrence rates with more than 10% positive samples indicate their importance. However, the clinical relevance for most of these species still remains to be elucidated. In this regard, we assumed that the colonization ability of individual species for the lung epithelia has an impact on the ecology of the CF respiratory tract, influence their microbial communities and finally the virulence potential of the bacterial assemblage. Such a concept of bacterial communities as one pathogenic entity in the context of CF was introduced recently by Rogers et al. [24]. The idea, however, of pathogenic bacterial assemblages to cause infections rather than single species has been raised previously [13]. To understand these colonization abilities, microbial community composition in individual samples was assessed and compared. The distribution in the MDS plot revealed a high diversity and high individuality of the microbial communities in the respiratory tract as assessed by molecular analyses (Fig. 1).

Long-term studies of the microbial community in a smaller cohort of CF patients similarly indicated the uniqueness of CF airway colonization in each patient [25]. A community consisting of the same bacteria even showed re-growth after antibiotic treatment rather than the immigration of new bacteria [38]. However, in each of the patients of our study at least one of the previously defined most prevalent species was observed, suggesting a major impact of these bacteria in the ecology of the CF airways. Hereby, the prevalent key pathogens *P*. *aeruginosa* and *S*. *aureus* were further observed to be also the most dominant species in terms of relative abundances in individual samples (Fig. 2). A positive relationship between prevalence and relative abundance of bacterial species was previously described for CF patients [26, 27]. This phenomenon is similarly known from other bacterial habitats and may be explained by a ‘simplistic model’ in which abundant bacteria are more likely to be detected whereas rare bacteria in one place are more likely below the detection limit in other locations [12]. However, such infrequently detected species may become more abundant in individual samples due to favourable environmental conditions. Interestingly, our NGS-based community profiling revealed dominance of specific bacteria in individual samples for *S*. *millerii*, *Nocardia* sp., *B*. *petrii*, *F*. *nucleatum* with higher relative abundances of these bacteria than the highly prevalent alpha-hemolytic Steptococci and *R*. *mucilaginosa*. Other known CF pathogens, like *A*. *xylosoxidans*, *S*. *maltophilia* and *B*. *cepacia* were observed with moderate relative abundances suggesting that the dominance of single species alone does not indicate their pathogenic potential but provides insights into their colonization abilities.

A strong correlation between the four most prevalent OTUs, such as *P*. *aeruginosa*, *S*. *aureus*, alpha-hemolytic *Streptococcus* and *R*. *mucilaginosa*, and clinical paramters, like age and lung function was not observed. However, certain tendency of less *P*. *aeruginosa*-dominated communities was observed with more diverse CFTR genotype. Studies with paediatric and adult CF patients similarly reported a correlation between the CFTR mutation and the microbial community, particularly an age-based correlation between the ΔF508-genotype and *P*. *aeruginosa* [28, 29]. A conceivable reason for this lack of correlation in our study might be the continuous treatment of our patient with antibiotics. A comparable reasoning could be applicable to the lack of a correlation with age and lung function (Figs. E and F in S1 File). On the other hand, our study did not include children and young adults. In the age range of 0–18 years lung function is decreasing by about 25% and until the age of 28 years by about 40% [30]. The given age distribution could mask significant statistical correlation with the bacterial community structure. A comparable effect could also mask the well described correlation between antibiotic treatment and abundances of *P*. *aeruginosa* [29]. In general, children do not need to be continuously treated with antibiotics and therefore, studies with juvenile cohorts could isolate the effect of antibiotic treatment and the occurrence of *P*. *aeruginosa* [29, 31]. Overall, this leads to the conclusion that the life history of individual CF patients and their lung microbiome needs to be considered if a complete understanding of the ecology and dynamics of these microbial communities is approached. These findings demonstrate the complexity of adult CF lung disease manifestation and illustrate that infections will only be understood considering polymicrobial aspects and individual history of the patient including the application of antibiotics.

A conceptual model of the lung microbiome of CF patients has been provided by the study of van der Gast et. al [27] dividing this bacterial community into core and satellite taxa. We would like to extend this model in that respect that the core community could consist of a single, dominant species as defined above. This species, be it a key pathogen or not, could act as an “attractor taxon” structuring the microenvironment of the CF lung for the satellite species. This concept would allow further systems biology analyses using meta-genomics and meta-transcriptomics studies resulting in a deterministic understanding of the ecology of the bacterial communities in the CF lung [32].

Dominant species (abundances of more than 50%) in communities could have a strong impact on the functioning of the CF airways, because these potentially pathogenic bacteria may play a major role in inter-species interaction including biofilm maturation and competition for finite resources [15]. Their success in colonization can be further estimated by their persistence in the airways. The ability to persistently colonize the respiratory tracts of CF airways, particularly with high relative abundances, may be regarded as an essential factor for bacterial virulence and pathogenicity. The key pathogens, *P*. *aeruginosa* and *S*. *aureus*, are becoming highly adapted on the clonal level to the human respiratory tract over time [22, 30] and therefore, constant communities with high relative abundances are not surprising for these bacteria. In the current study, however, such persistence was also observed for the anaerobic species *S*. *millerii* and *F*. *nucleatum* indicating good adaptation of these species to the associated environmental conditions in the respective patients. Therefore, they may also play a significant role in the ecology of CF airway communities and eventually influence the pathogenicity of the bacterial community. Particularly, species from the *S*. *millerii* group are thought to be underestimated infectious agents and observed co-colonization with *P*. *aeruginosa* has led to the hypothesis of polymicrobial interactions resulting in enhanced virulence in CF patients [33]. Accordingly, members of the *S*. *millerii* group were always detected together with *P*. *aeruginosa* in the current study.

Although, *F*. *nucleatum* is regularly isolated from healthy oral body sites, this species is known to be involved in serious infections with a strong ability to form co-aggregates with other species [34], indicating that the pathogenicity of bacterium might differ in different body parts and associated microbial communities. Similarly, *Nocardia* sp. is apparently more than a transient part of respiratory communities. Although infrequently detected in CF sputum samples [34], *Nocardia* sp. was the dominant bacteria in one community and was still present in a subsequent sputum sample 7 months later, indicating adaptive colonization abilities even for a rarely observed taxon. Studies on the biogeochemical role of these taxa could improve the understanding of the functioning of polymicrobial assemblages in the CF lung as demonstrated recently by a study including metatransciptome analyses [32].

### Conclusions

Overall, our study demonstrated the diversity of bacteria associated with the respiratory tracts of adult CF patients. Culture-independent methods like NGS analyses and SSCP fingerprints are useful methods to assess the community compositions and to identify emerging pathogens. Further studies on their clinical relevance are required to evaluate the need of their integration into future microbial diagnostics. Furthermore, microbial communities in CF patients are apparently highly individual and, therefore, the virulence of single species may similarly vary between different patients depending on the bacterial assemblages and the specific clones of established key taxa providing a highly specific microenvironment. These conditions may even vary across different areas of the lung since spatial heterogeneity in bacterial communities was observed in the CF lung [35]. In this respect, we suggest further studies on the role of the anaerobic bacteria of the *S*. *millerii* group as well as *F*. *nucleatum* to assess their contribution to tissue damage of CF airways. Antibiotic treatment, the current practice for adult CF patients, might be an overriding factor influencing the shaping forces in the life-long evolution of the lung microbiome [36]. Keeping these findings in mind, future studies with large CF cohorts should include sampling in different compartments of the CF airways and lung, and provide more detailed ecological information on the bacterial communities, including functional genomics and metabolic pathways of the bacteria by using whole genome sequencing and comparative metabolomics based on the biogeochemical network of the CF lung [32]. Ideally, longitudinal analyses of subgroups of patients with similar communities should allow a more comprehensive view on the impact of individual microbial communities and be complemented by a more detailed genetic assessment of the host and its clinical parameters to understand disease progression in cystic fibrosis.

## Materials and Methods

### Ethics statement

Ethical clearance for this study was obtained from the local ethics committee of the Medical School Hannover chaired by Prof. Tröger (at March 7, 2011; Reference number 1002–2011) and written informed consent was obtained from all study participants.

### Patient cohort and sample collection

Sputum samples were collected in sterile containers from 56 CF patients recruited in the CF outpatient clinic of the Hannover Medical School (MHH; Hannover, Germany), including a subgroup of 13 randomly chosen patients who provided sputum twice (n = 10) or three times (n = 3) within the sampling period of 2 years. In total, 72 separate sputum samples were collected after ethical approval for the current study was granted by the local health authority ethics committee. Sputum collection was done during the routine medical examination. The patients were between 18–51 years old and a median age of 31 years was calculated for the cohort. All patients were recruited in Northern Germany and were Caucasians. Both genders were equally represented (48.2% female and 51.8% male). Selection criteria for the CF patients were as follows: clinically stable condition, well characterised genetic status of the CFTR gene, about equal sex and adult (more than 18 years of age). Identification of CFTR-genotype was performed by polymerase chain reaction (PCR) and, if necessary, by sequencing of the amplicon according to the Guidelines for Molecular Diagnostics of the CF Society (*Leitlinie zur Molekulargenetischen Diagnostik der Cystischen Fibrose)* [39]. Pulmonary functions were measured as forced expiratory volume in 1 second (FEV_1_) with Ganshorn Body Scope version LF8.5E (Ganshorn Medizin Electronic; Niederlauer, Germany) before sputum was collected. No pulmonary exacerbation was observed by the time of sputum collection. Sputum samples were collected in duplicates and stored at -20°C. Application of antibiotics to the CF patients followed the recommendations of the International Standard of Care for adult CF patients [31, 37, 38]. In brief, the patients were under continuous treatment of antibiotics by inhalation applied twice a day at home. Inhalative antibiotics were mostly colistin or todramycin according to clinical needs. None of the patients received antibiotics intravenously during the time of study.

### Culture-based microbial diagnostics

Sputum samples for clinical microbial diagnostics were immediately processed in-house at the microbiological laboratory of the MHH. Routine cultivation methods were applied according to the German Quality Standards in Clinical Microbiology and Infectious Diseases [16].

### Sputum preparation and DNA extraction

An optimized protocol for the extraction of microbial DNA was developed by modifying the manufacture’s instructions of the GeneMATRIX kit (EurX Roboklon; Berlin, Germany). Briefly, sputum samples were aliquoted and boiled for 15 min. To decrease the viscosity, a cysteine buffer (2% NaOH, 1.45% sodium citrate and 0.5% N-acetylcysteine) was added in the same volume and the solution was mixed for 40 minutes. Water was added to a final volume of 15 ml and centrifuged for 30 min at 4000 g. Supernatant was discarded and the pellet resuspended in 300 µl lysis buffer (20 mM Tris-Cl with pH 8.0, 2 mM sodium EDTA, 1.2% Triton X-100). 6 mg of Lysozyme (Serva; Heidelberg, Germany) was added and incubated for 30 min at 37°C. To the total volume of the solution 0.5% of β-mercaptoethanol was given together with 50 U of Lyticase (Sigma-Aldrich; Steinheim, Germany). The solution was incubated for another 45 min at 37°C before centrifugation at 12.000 rpm for 10 min. The pellet was resuspended in 300 µl Lyse T buffer from GeneMATRIX Tissue & Bacteria DNA purification kit (EurX Roboklon; Berlin, Germany) and 20 µl of Proteinase K (Qiagen; Hilden, Germany) was added. After incubation of 2 hours at 56°C, the DNA was extracted by following the instructions of the manufactures. DNA extracts were kept frozen at -20°C for further analysis.

The described boiling of the sputum samples resulted in a higher DNA yield by about a factor of four. The bacterial composition and fingerprint patterns were comparable with or without boiling. More details on this DNA extraction procedure are given in the Supporting Information (Table D and Fig. K in S1 File).

### Fingerprints by SSCP electrophoresis and sequencing of individual bands

PCR amplification of parts of the rRNA gene from bacteria was performed using the primers 27F (5´-AGAGTTTGATCMTGGCTCAG-3’) and 521R (5’-ACCGTGGCTGCTGGCAC-3’) [17]. Amplicons of the 16S rRNA gene included the variable regions V1-V3 of the small subunit of the ribosome (SSU) with a amplicon size between 459 bp for *Nocardia* sp. and 505 bp for *Veillonella* sp. PCR was carried out using 50 ng DNA extracted from sputum in a final volume of 50 μl, starting with an initial denaturation for 15 min at 95°C. A total of 30 cycles (1 min at 95°C, 40 sec at 56°C, and 1 min at 72°C) was followed by a final elongation for 10 min at 72°C. 1.5 U of HotStarTaq DNA polymerase was used for all amplifications (Qiagen; Hilden, Germany). For single-strand DNA (ssDNA) preparation, reverse primer 521R was 5´-biotin labeled and magnetic streptavidin coated beads (Promega, Madison, Wis.) were applied to obtain ssDNA from the PCR amplicons according to Eichler et al. [18]. Dried pellets of ssDNA were resuspended in 7 µl of gel loading buffer (95% formamide, 10 mM NaOH, 0.25% bromphenol blue, 0.25% xylene cyanol). After incubation for 10 min at 95°C, the ssDNA samples were shortly stored on ice and loaded onto a non-denaturing polyacrylamide-like gel (0.6x MDE gel solution; Cambrex BioScience, Rockland, Maine) for SSCP electrophoresis. Molecular fingerprints of bacterial DNA were obtained at 20°C and 400 volt for 21 h. The gel was silver stained according to the method described by Bassam et al. [40]. Relative abundances were determined by the measurement of the intensity of the single bands in relation to the total intensity of all bands. SSCP gels were digitized using a HP Scanjet G4050 scanner. Bands with intensities of ≥5% of the total lane were considered for further statistical analysis. Intensities were determined by using the GelCompare II software (Applied Maths, Kortrijk, Belgium). More details on the SSCP fingerprints are given in Eichler et al. [41].

### High-throughput deep sequencing using the MiSeq platform of Illumina

PCR amplification of parts of the 16S rRNA gene, including the variable region V3, was performed by using the primers 341F (5´-ATTACCGCGGCTGCTGG-3’) and 518R (5’-CCTACGGGAGGCAGCAG-3’) [42]. Amplicon library preparation was performed according to Camarinha-Silva et al. [43]. Briefly, the 16S rRNA primers were designed with integrated complementary sequences to the Illumina specific adaptors to the 5’ends (Table E in S1 File). Further, a 6 nucleotide error correcting barcode (BC) was integrated in the forward primer, together with a 2 nucleotide CA linker to avoid amplification bias [44, 45]. PCR was carried out using 50 ng DNA of sputum extracts in a final volume of 50 μl, starting with an initial denaturation for 15 min at 95°C. A total of 20 cycles (10 sec at 98°C, 10 sec at 53°C, and 45 sec at 72°C) was followed by a final elongation for 2 min at 72°C. Only amplicons of the correct size were processed further. Negative controls were included in all PCR reactions as well as positive controls from *P*. *areuginosa* using DNA extracted from strain PAO1. Specific Illumina multiplexing sequencing primers as well as index primers were integrated with accordingly designed primers in a second subsequently performed PCR. The second PCR was carried out using 1 µl of the first PCR reaction as template. Additionally, 5 µl of Q solution (Qiagen; Hilden, Germany) were added to a final volume of 50 µl. Initial denaturation for 15 min at 95°C was followed by a total of 20 cycles (10 sec at 98°C, 10 sec at 62°C, and 45 sec at 72°C) and a final elongation for 2 min at 72°C. PCR products were separated on a 2% agarose gel and bands of correct size (around 330 bp) extracted and recovered using the QIAquick Gel Extraction kit (Qiagen; Hilden, Germany). Equimolar ratios of amplicons (50 ng) were pooled, all tagged with a unique combination of barcode and index. Quality of these libraries as well as the DNA concentration was measured by an Agilent Bioanalyzer. Libraries were sent for paired-end multiplex sequencing on a GAIIx Genome Analyzer (Deep, San Diego, CA). Image analysis and base calling were done by using the Deep Pipeline (version 1.7).

### Reproducibility of deep sequencing

DNA extraction and PCR amplification bias were estimated by subsets of sputum samples chosen to be analyzed repeatedly. From a subset of 8 sputum samples, DNA extractions were performed twice from different aliquots. From a subset of 10 samples, PCR amplicons and libraries were prepared twice and NGS performed in different runs. Sputum samples throughout these subsets exhibited different viscosity and visual appearance. Reproducibility was determined by comparison of the compositional data from each pair of subsets using the statistical RELATE routine analysis described below. Comparison for the different subsets revealed a high degree of agreement (Table A in S1 File).

### Data processing and phylogenetic analysis

Phylogenetic analysis of sequences was performed with the NCBI Tool BLAST/blastn and Ribosomal Data Base Project (RDP) Seqmatch Tool [46]. Closest taxonomic groups were determined by sequence similarity and running distance of ssDNA bands in the molecular fingerprints. For sequences obtained from SSCP fingerptints, species identification was defined for sequence similarity of ≥ 98%, ssDNA bands of same running distance were considered to be same species. Further, phylogenetic distance analyses were performed to confirm the identification of the individual operational taxonomic unit (OTU) (data not shown). For sequences obtained by Deep sequencing, only the forward end sequence reads were processed. A quality filter was applied and determination of representative reads were both done according to Camarinha-Silva et al. [43]. Only 90 bp of the forward 5’ end of all reads were used for further analysis, due to decreasing sequence quality. Representative reads were defined as i) present in at least one sample in a relative abundance > 1% of the total sequences of that sample, ii) present in at least 2% of samples at a relative abundance > 0.1% or iii) present in at least 5% of samples. A minimum read count of 2,000 sequences per sample were defined in the current study and previously shown to be sufficient to recapture the bacterial community [46]. Median number of total reads per sample was 10,933 sequences. For individual samples which were prepared and analyzed more than once, only the one with the most total sequence-reads was taken into account for the comparison of all 72 sputum samples. Species identification was defined for sequence similarity of ≥ 98% [47]. However, sequences with ambiguous results were defined according to the closest shared taxonomic level. Sequences affiliated to human DNA were removed before calculation of relative abundances of bacterial OTUs. Results from different Deep runs were pooled and a threshold of 0.5% relative abundance was chosen for each sample, in order to analyse only OTUs with a minimum relevance for the community. The relative abundance of individual OTUs in NGS was defined equal to the relative frequency of the associated bacterial sequences. In total, 65 OTUs from 72 sputum samples were identified and further processed (Table C in S1 File). A set of representative NGS-based sequences of the OTUs has been added to the Supporting Information (Table F in S1 File). The complete NGS data set is available in the European Nucleotide Archive (ENA) under the accession number PRJEB8060.

### Statistical analysis

Multivariant statistical analyses were performed using PRIMER 6 (Version.6.1.6, PRIMER-E, Plymouth Marine Laboratory, UK). Resemblance matrices were calculated using the Bray-Curtis similarity coefficient [48]. According to the similarities the samples were ordinated in non-metric multidimensional scaling (MDS) plots (with 50 random restarts), distances between samples in these plots reflect similarities of associated OTU compositions. To compare two resemblance matrices, the RELATE routine analysis was applied. This comparative (Mantel-type) test measures the similarity and calculates the Spearman's rank correlation coefficient rho [49]. Similarity is measured by Spearman’s rho and data sets were permuted [50]. A perfect Spearman correlation would give the values of +1 or −1. For all analyses the number of permuted statistics greater than or equal to rho value of the actual sample was zero.

## Supporting Information

S1 File This file contains Tables A to F and Figs. A to K. **Table A**, **Comparison of data sets from bacterial community profiling.** Resemblance matrices were calculated for each dataset and compared using spearman rank correlation (rho). Major discrepancies observed for each pair of data sets are mentioned. For the comparison of SSCP fingerprinting and deep sequencing, only OTUs with a relative abundance of ≥ 5% were considered for this statistical comparison. Similarity was measured by Spearman’s rho and data sets are permuted. **Table B, Summary of parameters characterizing the adult cohort of CF patients and the alpha diversity of the bacterial communities.** Richness and Shannon diversity index were calculated based on the relative abundances of single OTUs based on NGS sequence reads as detailed in the Materials and Methods. **Table C, OTUs observed with deep sequencing exhibiting a minimum relative abundance of 0.5%.** Sequence similarity revealed by BLAST is given as well as the closest representative (accession number and name) for each OTU. Comparison with SSCP fingerprinting allowed phylogenetic affiliation of some previously ambiguous OTUs to species level. Streptococcus species identified by SSCP are mentioned with sequence identity scores in percent as well as accession numbers of closest representatives in the end of the table. For Illumina-based data, number of positive samples for each OTU is given as well as minimum and maximum relative abundances in individual samples. **Table D, DNA retrieved from 300µl CSF sputum after extraction from boiled vs non-boiled samples (cellular fraction retrieved from the pellet). Table E, Primers used for library preparation in Illumina-based sequencing.** Underlined letters denote complementary sequences for the variable region V3 of the 16S rRNA gene. Eleven different barcodes were used, each indicated with bold letters within the primer sequence. In a second PCR, amplicons for libraries were accomplished by adding Illumina-specific indices. **Table F, Consensus sequences of for all major OTUs obtained by NGS sequencing of the 16S rRNA gene**. **Fig. A., Comparison of prevalence of bacteria in the cohort observed by deep sequencing (NGS, white bars) and SSCP fingerprinting (black bars).** OTUs were identified to the genus level and listed accordingly on the left. Streptococci were additionally distinguished between alpha-hemolytic streptococci (*Streptococcus*) and the *Streptococcus millerii* group. For each diagnostic method, only OTUs with ≥ 5% relative abundance were taken into account for this comparison. **Fig. B, Phylogeny of detected *Streptococcus* species in sputum samples.** High sequence variation was observed in bands from SSCP fingerprints. Closest described representatives from databases are given in the taxonomic tree. Associated OTUs defined by deep sequencing are indicated with brackets. Aerobic alpha-hemolytic streptococci are further marked with bold lines. *Staphylococcus aureus* was included for rooting of the tree. Scale bar represents base substitution per site. **Fig. C, Comparisons of the relative abundance of the most dominant OTU in each individual sample assessed with deep sequencing (NGS, white bars) and SSCP fingerprinting (black bars).** Sputum samples are labelled by individual numbers and mentioned on the left. The samples are sorted by decreasing similarity of the relative abundances of OTUs between the two molecular methods. **Fig. D, Correlation of richness with Shannon diversity index based on the NGS sequence abundance data of all 56 patients (n = 56).** Richness and Shannon diversity index are calculated based on the relative abundances of single OTUs based on NGS sequence reads as detailed in the Materials and Methods. The logarithmic regression provided the best fit to the data set. A comparable logarithmic correlation was observed when the data of the longitudinal observations were included (n = 72). **Fig. E, Comparison of lung function with Shannon diversity index of all CF patients (n = 55).** The Shannon diversity was calculated as in indicated in Fig. D in S1 File. Standard linear and non-linear correlation analyses did not show a clear relationship. **Fig. F, Comparison of age with Shannon diversity index of all CF patients (n = 56).** The Shannon diversity was calculated as in indicated in Fig. D in S1 File. Standard linear and non-linear correlation analyses did not show a clear relationship. **Fig. G, Comparison between age of patients (white bars) and relative abundance of *P*. *aeruginosa* (black bars).** Samples are sorted by age of the patient at the individual time point of sputum collection. Age of patient is given in years and relative abundance of *P*. *aeruginosa* is given in % on y-axis. For each patient, both parameters are indicated. Missing bars for *P*. *aeruginosa* indicate its absence in the sample. A median relative abundance of 24.8% was calculated for the bacteria. Median age at time point of sputum collection was 31 years. **Fig. H, MDS plot from bacterial community composition observed in sputum samples with superimposed antibiotic treatment.** Six categories of antibiotic treatment were defined and are indicated with individual symbols. Antibiotics were given by inhalation (inh.), orally (p.o.), or intravenously (i.v.) within past 14 days before sampling of sputum or by none of these categories (other). MDS plot is based on the respective plots in Fig. 3. **Fig. I, Comparison between relative abundance of *P*. *aeruginosa* (grey bars) and lung function of the patient (black bars).** Samples are sorted by relative abundance of *P*. *aeruginosa*. Lung functions of the patients at the individual time point of sputum collection is measured by the predicted FEV_1_ value. Both, relative abundance of *P*. *aeruginosa* and lung function are given in % on y-axis. For each patient, both parameters are indicated. Missing bars for *P*. *aeruginosa* indicate its absence in the sample and missing bars for FEV_1_ indicates no measurement for the patient at time point of sputum collection. A median FEV_1_ of 35% was calculated for the cohort. **Fig. J, Dynamics of community compositions from 13 CF patients from which sputum samples were obtained twice or three times.** Each OTU is indicated by a specific colour and further defined in the legend. CF patients are identified by numbers and sputum samples are shown in chronological order, hereby, time intervals to the initial sample are indicated in the figure. **Fig. K, 16S rRNA gene based community fingerprint by SSCP for boiled (B1, B2, B3) vs. non-boiled samples (U1, U2, U3).** B1 and U1 in the right part of the gel was a repetition of the single strand preparation. (St = Standard using 5 different bacterial species).
(DOC)
