# Supplementary material for: High Individuality of Respiratory Bacterial Communities in a Large Cohort of Adult Cystic Fibrosis Patients under Continuous Antibiotic Treatment
Source: PLoS One. 2015 Feb 11;10(2):e0117436. doi: 10.1371/journal.pone.0117436 (PMC4324987; doi:10.1371/journal.pone.0117436)
Supplement: S1 File — Table A, Comparison of data sets from bacterial community profiling. Resemblance matrices were calculated for each dataset and compared using spearman rank correlation (rho). Major discrepancies observed for each pair of data sets are mentioned. For the comparison of SSCP fingerprinting and deep sequencing, only OTUs with a relative abundance of ≥ 5% were considered for this statistical comparison. Similarity was measured by Spearman’s rho and data sets are permuted. Table B, Summary of parameters characterizing the adult cohort of CF patients and the alpha diversity of the bacterial communities. Richness and Shannon diversity index were calculated based on the relative abundances of single OTUs based on NGS sequence reads as detailed in the Materials and Methods. Table C, OTUs observed with deep sequencing exhibiting a minimum relative abundance of 0.5%. Sequence similarity revealed by BLAST is given as well as the closest representative (accession number and name) for each OTU. Comparison with SSCP fingerprinting allowed phylogenetic affiliation of some previously ambiguous OTUs to species level. Streptococcus species identified by SSCP are mentioned with sequence identity scores in percent as well as accession numbers of closest representatives in the end of the table. For Illumina-based data, number of positive samples for each OTU is given as well as minimum and maximum relative abundances in individual samples. Table D, DNA retrieved from 300µl CSF sputum after extraction from boiled vs non-boiled samples (cellular fraction retrieved from the pellet). Table E, Primers used for library preparation in Illumina-based sequencing. Underlined letters denote complementary sequences for the variable region V3 of the 16S rRNA gene. Eleven different barcodes were used, each indicated with bold letters within the primer sequence. In a second PCR, amplicons for libraries were accomplished by adding Illumina-specific indices. Table F, Consensus sequences of for all major O [file pone.0117436.s001.doc]

**SUPPorting Information S1 File**

**High Individuality of Respiratory Bacterial Communities in a Large Cohort of Adult Cystic Fibrosis Patients under Continuous Antibiotic Treatment**

***Revised version January 20, 2015***

Rolf Kramer^1^, Annette Sauer-Heilborn^2^, Tobias Welte^2,4^, Ruy Jauregui^3^, Ingrid Brettar^1^, Carlos A. Guzman^1^, Manfred G. Höfle^1^*

1 Department of Vaccinology and Applied Microbiology, Helmholtz Centre for Infection Research, Braunschweig, Germany; 2 Department of Pneumology, Hannover Medical School, Hannover, Germany; 3 Department of Medical Microbiology, Helmholtz Centre for Infection Research, Braunschweig, Germany; 4 German Centre for Infection Research and German Centre for Lung Research, Hannover, Germany

**22 pages, 6 Tables and 11 Figures**

*Corresponding author: Manfred G. Höfle, E-Mail: manfred.hoefle@helmholtz-hzi.de

| **Data sets compared** | **Spearman rho** | **Major discrepancies** |
| --- | --- | --- |
| Illumina; different extractions | 0.911 | mean of 2.9% differences in OTU abundances |
| Illumina; different sequencing reactions | 0.976 | mean of 3.5% differences in OTU abundances |
| Illumina and SSCP fingerprinting; all samples (≥ 5%) | 0.816 | different taxonomic resolution for the genus *Streptococcus* |

**Table A.** **Comparison of data sets from bacterial community profiling.** Resemblance matrices were calculated for each dataset and compared using spearman rank correlation (rho). Major discrepancies observed for each pair of data sets are mentioned. For the comparison of SSCP fingerprinting and deep sequencing, only OTUs with a relative abundance of ≥ 5% were considered for this statistical comparison. Similarity was measured by Spearman’s rho and data sets are permuted.

| **parameter** |  | **average (n=56)** | **+SD** | **range** |
| --- | --- | --- | --- | --- |
|  |  |  |  |  |
| Age |  | 31.33 | 8.37 | 18 – 51 |
| FEV_1_ value (lung function) % |  | 41.09 | 22.36 | 18 - 114 |
| Richness |  | 8.59 | 4.79 | 1 - 22 |
| Shannon diversity index |  | 1.13 | 0.53 | 0.07 – 2.03 |

**Table B. Summary of parameters characterizing the adult cohort of CF patients and the alpha diversity of the bacterial communities.** Richness and Shannon diversity index were calculated based on the relative abundances of single OTUs based on NGS sequence reads as detailed in the Materials and Methods.

| **OTU Illumina** | **Identity in %** | **closest representative** | **species by SSCP** | **pos. samples (total no.)** | **min. abund. in %** | **max. abund. in %** |
| --- | --- | --- | --- | --- | --- | --- |
| Streptococcus -1 | 100 | KC632197.1 Streptococcus parasanguinis |  | 63 | 0.86 | 51.67 |
| Rothia mucilaginosa | 100 | KC632201.1Rothia mucilaginosa | 99% R. mucilaginosa DQ409140.1 | 60 | 0.54 | 50.06 |
| Pseudomonas -1 | 100 | HF572851.1 Pseudomonas aeruginosa | 100% P. aeruginosa  CP003149.1 | 57 | 0.63 | 93.31 |
| Rothia -2 | 100 | KC632226.1 Rothia dentocariosa |  | 36 | 0.72 | 25.77 |
| Granulicatella -1 | 100 | JF803551.1 Granulicatella para-adiacens |  | 33 | 0.53 | 5.87 |
| Prevotella -1 | 100 | JN867317.1 Prevotella melaninogenica |  | 30 | 0.55 | 38.32 |
| Staphylococcus -1 | 100 | KC465401.1 Staphylococcus aureus | 99% S. aureus AB680391.1 | 27 | 0.60 | 87.81 |
| Gemella haemolysans | 100 | HE974940.1 Gemella haemolysans | 100% G.haemolysans NR025903.1 | 22 | 0.57 | 31.49 |
| Gemella -2 | 100 | AY757357.1 Gemella sanguinis |  | 22 | 0.51 | 4.28 |
| Atopobium -1 | 100 | GU425926.1 Atopobium rimae |  | 18 | 0.55 | 3.23 |
| Streptococcus -3 | 100 | KC632205.1 Streptococcus sanguinis |  | 18 | 0.54 | 8.77 |
| Streptococcus millerii | 100 | KC632214.1 Streptococcus anginosus |  | 17 | 0.54 | 75.53 |
| Streptococcus -2 | 99 | KC632193.1 Streptococcus salivarius |  | 16 | 0.60 | 10.10 |
| Actinomyces graevenitzii | 97 | AY866429.1 Actinomyces graevenitzii |  | 16 | 0.58 | 14.51 |
| Actinomyces -1 | 100 | HQ850579.1 Actinomyces turicensis |  | 15 | 0.57 | 14.16 |
| Actinomyces -2 | 97/100 | HQ616392.1 Actinomyces sp. / JQ031125.1 Actinomyces funkei |  | 13 | 0.54 | 5.53 |
| Veillonella atypica | 100 | KC632238.1 Veillonella dispar |  | 13 | 0.52 | 13.83 |
| Veillonella -2 | 100 | HM596287.1 Veillonella sp. |  | 11 | 0.70 | 5.98 |
| Fusobacterium nucleatum | 100 | NR_074412.1 Fusobacterium nucleatum | 98% F.nucleatum AB588016.1 | 11 | 0.55 | 53.52 |
| Achromobacter -1 | 100 | KC010531.1 Achromobacter xylosoxidans | 99% A. xylosoxidans EU266588.1 | 6 | 0.77 | 46.35 |
| Stenotrophomonas maltophilia | 100 | KC683776.1 Stenotrophomonas maltophilia | 99% S.maltophilia JX848739.1 | 6 | 0.62 | 15.41 |
| Mogibacterium -1 | 100 | KC632200.1 Mogibacterium diversum |  | 5 | 0.51 | 1.31 |
| Actinomyces -3 | 97/100 | HQ616392.1 Actinomyces sp. / JQ031122.1 Actinomyces funkei |  | 5 | 0.66 | 1.74 |
| TM7 | 100 | GQ422738.1 TM7 phylum sp. Oral |  | 5 | 0.52 | 1.93 |
| Porphyromonas -1 | 100 | GU409265.1 Porphyromonas sp. |  | 4 | 0.76 | 1.58 |
| Bifidobacterium longum | 100 | HE974928.1 Bifidobacterium longum |  | 4 | 0.54 | 3.55 |
| Stomatobaculum longum | 100 | HM120209.1 Stomatobaculum longum |  | 4 | 0.51 | 4.36 |
| Clostridiaceae -1 | 100 | NR_074652.1 Clostridium phytofermentans |  | 4 | 0.50 | 1.11 |
| Prevotella pallens | 100 | KC632223.1 Prevotella pallens |  | 4 | 0.55 | 0.90 |
| Nocardia -1 | 100 | KC478309.1 Nocardia farcinica |  | 3 | 1.10 | 61.87 |
| Solobacterium moorei | 100 | JX104033.1 Solobacterium sp. |  | 3 | 0.57 | 0.70 |
| Actinomyces -4 | 96/95 | EF473992.1 Actinomyces sp. / JF803550.1 Actinomyces graevenitzii |  | 3 | 3.36 | 13.04 |
| Prevotella salivae | 100 | JN867237.1 Prevotella salivae |  | 3 | 0.52 | 1.58 |
| Granulicatella elegans | 100 | JN801174.1 Granulicatella elegans |  | 3 | 0.60 | 0.81 |
| Streptococcus -5 | 100 | GU326246.1 Streptococcus pneumoniae |  | 3 | 0.74 | 1.17 |
| Actinomyces -5 | 100 | JX524818.1 Actinomyces oris |  | 3 | 0.53 | 0.75 |
| Lactobacillus -2 | 100 | KC456368.1 Lactobacillus fermentum |  | 3 | 0.60 | 1.40 |
| Prevotella - 2 | 100 | JN867292.1 Prevotella oris |  | 2 | 5.64 | 12.27 |
| Rothia-3 | 100 | GQ900845.1 Rothia sp. |  | 2 | 0.51 | 1.10 |
| Haemophilus -2 | 100 | JF506651.1 Haemophilus parainfluenzae |  | 2 | 1.21 | 1.21 |
| Streptoccocus -4 | 99 | KC632199.1 Streptococcus australis |  | 2 | 0.51 | 0.67 |
| Veillonella -4 | 100 | AB679112.1 Veillonella tobetsuensis |  | 2 | 1.52 | 2.04 |
| Megasphaera micronuciformis | 98.59 | JF803576.1 Megasphaera micronuciformis |  | 2 | 0.50 | 0.83 |
| Porphyromonas -2 | 100 | JF803575.1 Porphyromonas sp. |  | 2 | 1.05 | 2.49 |
| Atopobium -1 | 100 | KC297229.1 Atopobium parvulum |  | 2 | 0.54 | 1.29 |
| Prevotella tannerae | 100 | GU561345.1 Prevotella tannerae |  | 2 | 0.52 | 1.26 |
| Burkholderia -1 | 100 | HF678361.1 Burkholderia cepacia | 100% B. cepacia AB680641.1 | 1 | 8.60 | 8.60 |
| Micrococcineae -1 | 100 | KC346300.1 Streptomyces violaceorectus |  | 1 | 1.12 | 1.12 |
| Neisseria -2 | 100 | KC178474.1 Neisseria mucosa |  | 1 | 2.43 | 2.43 |
| Acinetobacter -1 | 100 | KC337241.1| Acinetobacter tjernbergiae |  | 1 | 1.09 | 1.09 |
| Neisseria flavescens | 100 | KC178511.1 Neisseria flavescens |  | 1 | 1.61 | 1.61 |
| Bordetella petrii | 100 | HE672085.1 Bordetella petrii |  | 1 | 55.88 | 55.88 |
| Prevotella -3 | 100 | GU409603.1 Prevotella sp. |  | 1 | 2.92 | 2.92 |
| Veillonella -3 | 93 | KC632234.1 Veillonella sp. |  | 1 | 1.94 | 1.94 |
| Scardovia wiggsiae | 100 | HM596282.1 Scardovia wiggsiae |  | 1 | 0.54 | 0.54 |
| Prevotella nanceiensis | 100 | JN867319.1 Prevotella nanceiensis |  | 1 | 0.68 | 0.68 |
| Capnocytophaga gingivalis | 100 | GU561334.1 Capnocytophaga gingivalis |  | 1 | 1.81 | 1.81 |
| Leptotrichia wadei | 100 | AB588021.1 Leptotrichia wadei |  | 1 | 0.53 | 0.53 |
| Lactobacillus kalixensis | 100 | FR683096.1 Lactobacillus kalixensis |  | 1 | 1.39 | 1.39 |
| Prevotella -4 | 98 | GU413291.1 Prevotella sp. |  | 1 | 2.28 | 2.28 |
| Alloscardovia -1 | 100/97 | AB425070.1 Alloscardovia sp. / AB437351.1 Bifidobacterium psychraerophilum |  | 1 | 1.90 | 1.90 |
| Propionibacterium acidifaciens | 100 | JF803561.1 Propionibacterium acidifaciens |  | 1 | 0.60 | 0.60 |
| Haemophilus influenzae | 100 | CP000057.2 Haemophilus influenzae |  | 1 | 1.11 | 1.11 |
| Leptotrichia -2 | 100 | NR_074440.1 Leptotrichia buccalis |  | 1 | 0.74 | 0.74 |
| Propionibacterium acnes | 100 | JQ435685.1 Propionibacterium acnes |  | 1 | 3.59 | 3.59 |
| *only in SSCP* |  |  | 99% S. parasanguinis AY281078 |  |  |  |
| *only in SSCP* |  |  | 100% S. salivarius FR873482.1 |  |  |  |
| *only in SSCP* |  |  | 99% S. mitis FN568063.1 |  |  |  |
| *only in SSCP* |  |  | 99% S. gordonii AY281088.1 |  |  |  |
| *only in SSCP* |  |  | 100% S. sanguinis AB596946.1 |  |  |  |
| *only in SSCP* |  |  | 99% S. peroris GU425263.1 |  |  |  |
| *only in SSCP* |  |  | 99% S. oralis AF003932 |  |  |  |
| *only in SSCP* |  |  | 99% S. anginosus AF306838.1 |  |  |  |
| *only in SSCP* |  |  | 100% S. intermedius AF104671.1 |  |  |  |

**Table C. OTUs observed with deep sequencing exhibiting a minimum relative abundance of 0.5%.** Sequence similarity revealed by BLAST is given as well as the closest representative (accession number and name) for each OTU. Comparison with SSCP fingerprinting allowed phylogenetic affiliation of some previously ambiguous OTUs to species level. Streptococcus species identified by SSCP are mentioned with sequence identity scores in percent as well as accession numbers of closest representatives in the end of the table. For Illumina-based data, number of positive samples for each OTU is given as well as minimum and maximum relative abundances in individual samples.

**DNA extraction of sputum samples using boiling**

We compared boiling and non-boiling of CF sputum samples for the extraction of DNA. For this experiment we pooled 2 g of original sputum from 5 CF patients (a subset of the cohort studied which had been stored frozen at -20°C) and homogenized the material. This master sample was aliquoted to six samples, 300 µl each. Three samples were submitted to the DNA extraction procedure with boiling, three were not boiled. The DNA extraction procedure was exactly the one described in the manuscript: three replicates were boiled in the way described, three replicates were not boiled. The DNA content of the cellular fraction (retrieved as pellet) and in the supernatant was quantified using a Nanodrop photometer. The following results for the DNA yield of the cellular fraction of boiled vs non-boiled samples were obtained using a Nanodrop photometer:

| | sample no. | ng DNA/µl (eluted in 50µl) | ratio 260/280 nm | DNA yield  (µg DNA/g sputum) | mean DNA yield (SD) for 3 replicates | | --- | --- | --- | --- | --- | | B1- boiled | 433 | 1.95 | 78.5 | 68.4 (16.3) | | B2- boiled | 339 | 1.94 | 49.6 | | B3- boiled | 421 | 1.94 | 77.2 | | U1 - no boiling | 79 | 1.87 | 19.2 | 17.7 (1.9) | | U2 - no boiling | 85 | 1.89 | 15.5 | | U3 - no boiling | 92 | 1.88 | 18.4 | |
| --- | --- | --- | --- | --- | --- | --- | --- | --- | --- | --- | --- | --- | --- | --- | --- | --- | --- | --- | --- | --- | --- | --- | --- | --- | --- | --- | --- | --- | --- | --- | --- |

**Table D.** **DNA retrieved from 300µl CSF sputum after extraction from boiled vs non-boiled samples (cellular fraction retrieved from the pellet).**

The DNA yield of the cellular fraction (retrieved as pellet) was almost four times as high (3.9 x) for the boiled samples than for the unboiled samples (see Table 1). This phenomenon was observed and explained in former studies (Homes & Quigley 1981) in a way that boiling generates clots composed of partially denatured DNA with proteins that easily can be precipitated. The high content of DNA and protein (mostly mucines) in CF sputum as demonstrated earlier by Brandt et al. (1995) may have contributed to this clot formation. The DNA content of the supernatant ranged from a mean of 6.8 mg DNA/g sputum (SD 1.1mg/g) in the boiled samples to 9.0 mg DNA/g sputum (SD 1.2 mg/g) in the unboiled samples. Most of the DNA in the supernatant can be considered as extracellular DNA as already shown by Brandt et al. (1995) and is well within the range of up to 9.5 mg/g sputum as described in their study. The high content of extracellular DNA is a typical feature of CF sputum partially responsible for the high viscosity and suggested to be treated by application of recombinant human DNase (Shak et al. 1990, Brandt et al. 1995).

***Comparison of boiled vs the non-boiled DNA extracts by SSCP Community fingerprints***

16S rRNA gene-based community fingerprints using SSCP electrophoresis were generated as described in the manuscript based on the cellular DNA fraction. These fingerprints were used to get an overview on the effect of boiling in terms of bacteria species composition and diversity of the samples analyzed. The community fingerprint showed identical patterns for boiled and non-boiled samples as shown in **Figure K**. We concluded that the boiling of sputum samples prior to the applied commercial extraction technology resulted in a higher DNA retrieval from sputum of CF patients. The determination of the community pattern and composition was not changed by using either boiled or non-boiled samples. Therefore, we have included the boiling step in our standard DNA extraction protocol for the molecular analyses of the CF sputum samples.

| forward primers | sequence (5'->3') |
| --- | --- |
| V3_1 | ACACTCTTTCCCTACACGACGCTCTTCCGATCT**AAGCCT**CAATTACCGCGGCTGCTGG |
| V3_2 | ACACTCTTTCCCTACACGACGCTCTTCCGATCT**AAGTTA**CAATTACCGCGGCTGCTGG |
| V3_3 | ACACTCTTTCCCTACACGACGCTCTTCCGATCT**AATACG**CAATTACCGCGGCTGCTGG |
| V3_4 | ACACTCTTTCCCTACACGACGCTCTTCCGATCT**AATCGA**CAATTACCGCGGCTGCTGG |
| V3_5 | ACACTCTTTCCCTACACGACGCTCTTCCGATCT**AATGAT**CAATTACCGCGGCTGCTGG |
| V3_6 | ACACTCTTTCCCTACACGACGCTCTTCCGATCT**ACCAAT**CAATTACCGCGGCTGCTGG |
| V3_7 | ACACTCTTTCCCTACACGACGCTCTTCCGATCT**ACCGTC**CAATTACCGCGGCTGCTGG |
| V3_8 | ACACTCTTTCCCTACACGACGCTCTTCCGATCT**ACCTCG**CAATTACCGCGGCTGCTGG |
| V3_9 | ACACTCTTTCCCTACACGACGCTCTTCCGATCT**ACGAGG**CAATTACCGCGGCTGCTGG |
| V3_10 | ACACTCTTTCCCTACACGACGCTCTTCCGATCT**ACTATA**CAATTACCGCGGCTGCTGG |
| V3_11 | ACACTCTTTCCCTACACGACGCTCTTCCGATCT**ACTTAC**CAATTACCGCGGCTGCTGG |
| reverse primer | sequence (5'->3') |
| IlluRevAdap_V3 | GTGACTGGAGTTCAGACGTGTGCTCTTCCGATCTCCTACGGGAGGCAGCAG |

**Table E. Primers used for library preparation in Illumina-based sequencing.** Underlined letters denote complementary sequences for the variable region V3 of the 16S rRNA gene. Eleven different barcodes were used, each indicated with bold letters within the primer sequence. In a second PCR, amplicons for libraries were accomplished by adding Illumina-specific indices.

**Figure A. Comparison of prevalence of bacteria in the cohort observed by deep sequencing (NGS, white bars) and SSCP fingerprinting (black bars).** OTUs were identified to the genus level and listed accordingly on the left. Streptococci were additionally distinguished between alpha-hemolytic streptococci (*Streptococcus*) and the *Streptococcus millerii* group. For each diagnostic method, only OTUs with ≥ 5% relative abundance were taken into account for this comparison.

**Figure B.** **Phylogeny of detected *Streptococcus* species in sputum samples.** High sequence variation was observed in bands from SSCP fingerprints. Closest described representatives from databases are given in the taxonomic tree. Associated OTUs defined by deep sequencing are indicated with brackets. Aerobic alpha-hemolytic streptococci are further marked with bold lines. *Staphylococcus aureus* was included for rooting of the tree. Scale bar represents base substitution per site.

**Figure C.** **Comparisons of the relative abundance of the most dominant OTU in each individual sample assessed with deep sequencing (NGS, white bars) and SSCP fingerprinting (black bars).** Sputum samples are labelled by individual numbers and mentioned on the left. The samples are sorted by decreasing similarity of the relative abundances of OTUs between the two molecular methods.

**Figure D. Correlation of richness with Shannon diversity index based on the NGS sequence abundance data of all 56 patients (n=56).** Richness and Shannon diversity index are calculated based on the relative abundances of single OTUs based on NGS sequence reads as detailed in the Materials and Methods. The logarithmic regression provided the best fit to the data set. A comparable logarithmic correlation was observed when the data of the longitudinal observations were included (n=72).

**Figure E. Comparison of lung function with Shannon diversity index of all CF patients (n=55).** The Shannon diversity was calculated as in indicated in **Figure S4.** Standard linear and non-linear correlation analyses did not show a clear relationship.

**Figure F. Comparison of age with Shannon diversity index of all CF patients (n=56).** The Shannon diversity was calculated as in indicated in **Figure S4**. Standard linear and non-linear correlation analyses did not show a clear relationship.

. **Figure G.** **Comparison between age of patients (white bars) and relative abundance of *P. aeruginosa* (black bars).** Samples are sorted by age of the patient at the individual time point of sputum collection. Age of patient is given in years and relative abundance of *P. aeruginosa* is given in % on y-axis. For each patient, both parameters are indicated. Missing bars for *P. aeruginosa* indicate its absence in the sample. A median relative abundance of 24.8 % was calculated for the bacteria. Median age at time point of sputum collection was 31 years.


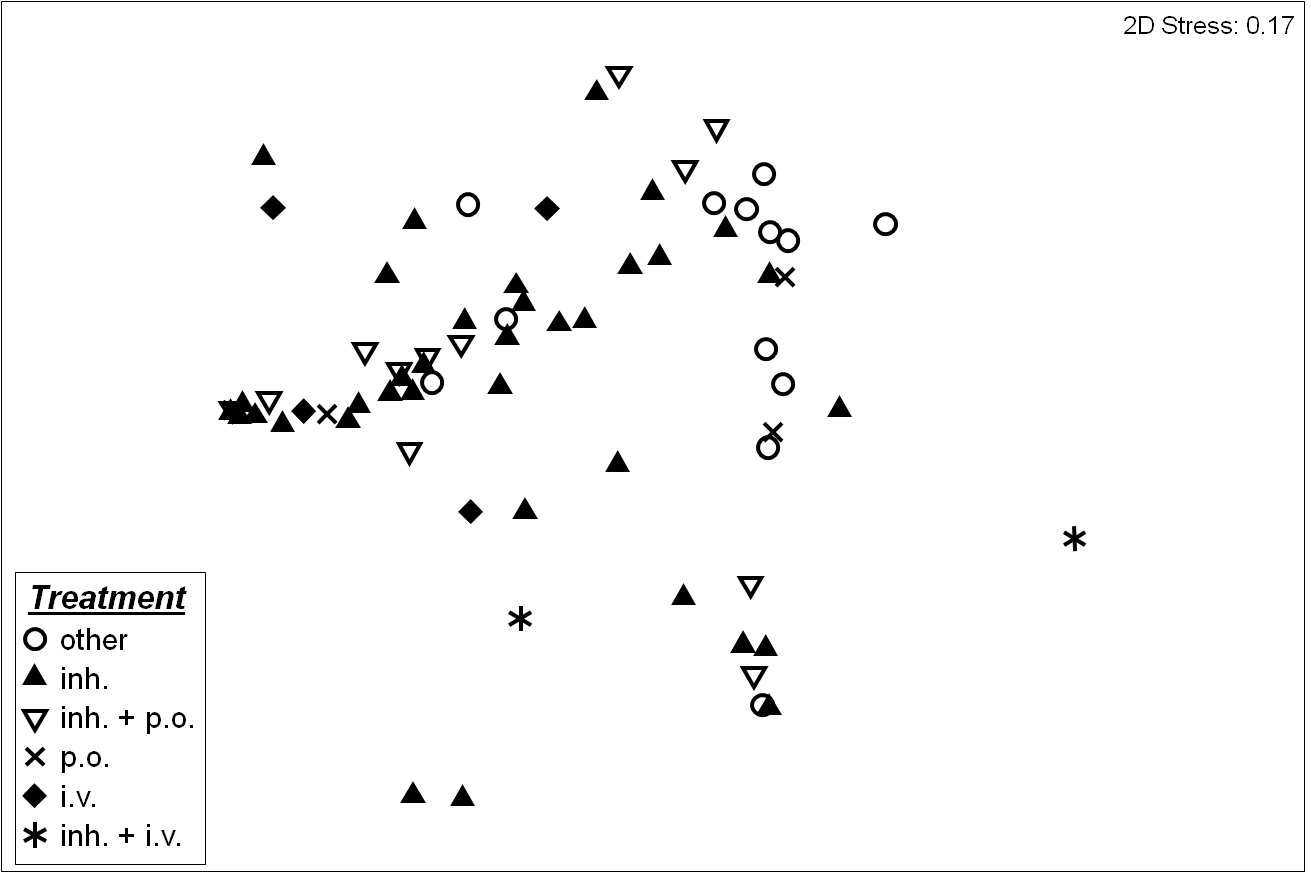


**Figure H. MDS plot from bacterial community composition observed in sputum samples with superimposed antibiotic treatment.** Six categories of antibiotic treatment were defined and are indicated with individual symbols. Antibiotics were given by inhalation (inh.), orally (p.o.), or intravenously (i.v.) within past 14 days before sampling of sputum or by none of these categories (other). MDS plot is based on the respective plots in Figure 3.

**Figure I.** **Comparison between relative abundance of *P. aeruginosa* (grey bars) and lung function of the patient (black bars).** Samples are sorted by relative abundance of *P. aeruginosa*. Lung functions of the patients at the individual time point of sputum collection is measured by the predicted FEV_1_ value. Both, relative abundance of *P. aeruginosa* and lung function are given in % on y-axis. For each patient, both parameters are indicated. Missing bars for *P. aeruginosa* indicate its absence in the sample and missing bars for FEV_1_ indicates no measurement for the patient at time point of sputum collection. A median FEV_1_ of 35 % was calculated for the cohort.

**Figure J. Dynamics of community compositions from 13 CF patients from which sputum samples were obtained twice or three times.** Each OTU is indicated by a specific colour and further defined in the legend. CF patients are identified by numbers and sputum samples are shown in chronological order, hereby, time intervals to the initial sample are indicated in the figure.


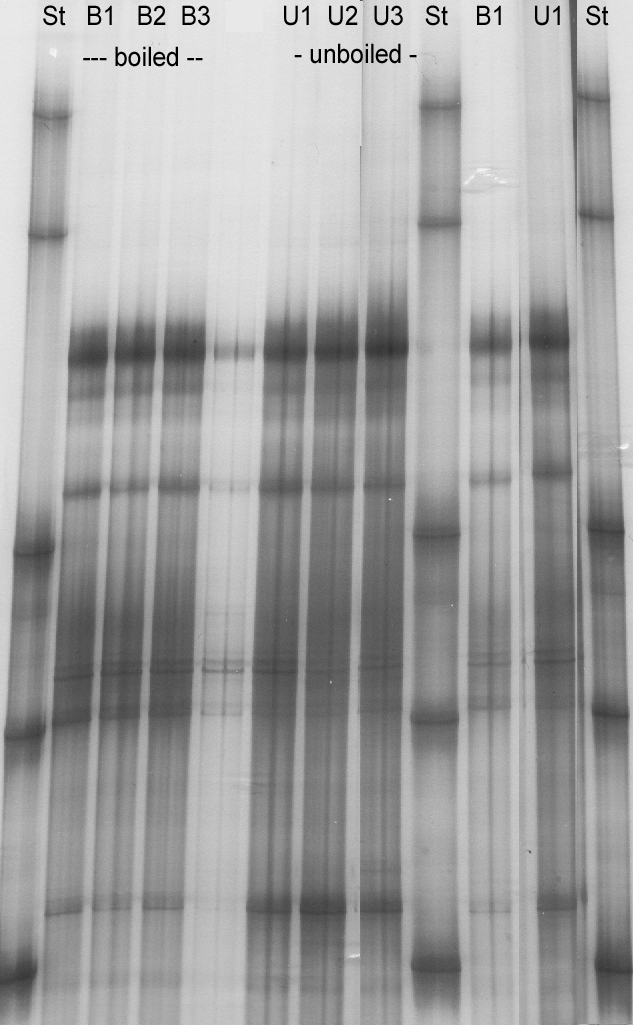


**Figure K. 16S rRNA gene based community fingerprint by SSCP for boiled (B1, B2, B3) vs. non-boiled samples (U1, U2, U3).** B1 and U1 in the right part of the gel was a repetition of the single strand preparation. (St = Standard using 5 different bacterial species).

**References Supporting Information**

T Brandt, S Breitenstein, H von der Hardt, B Tümmler (1995). DNA concentration and length in sputum of patients with cystic fibrosis during inhalation with recombinant human DNase.

Thorax 50: 880-882.

DS Holmes, M Quigley (1981). A rapid boiling method for the preparation of bacterial plasmids. Analytical biochemistry, 114: 193-197.

Shak S, Capon DJ, Hellmiss R, Marsters SA, Baker CL (1990). Recombinant human DNase I reduces the viscosity of cystic fibrosis sputum. Proc Natl Acad Sci USA, 87: 9188–9192.
